# Common and Distinct Neural Substrates for Approach- and Avoidance-Motivated Actions

**DOI:** 10.1523/JNEUROSCI.0238-26.2026

**Published:** 2026-07-30

**Authors:** Sho K. Sugawara, Yoshihisa Nakayama, Yuki H. Hamano, Tetsuya Yamamoto, Masaki Fukunaga, Norihiro Sadato, Yukio Nishimura

**Affiliations:** ^1^Neural Prosthetics Project, Tokyo Metropolitan Institute of Medical Science, Setagaya, Tokyo 156-8506, Japan; ^2^Division of Cerebral Integration, National Institute for Physiological Sciences, Okazaki, Aichi 444-8585, Japan; ^3^Department of Liberal Arts and Basic Sciences, College of Industrial Technology, Nihon University, Narashino, Chiba 275-8576, Japan; ^4^Research Institute for Science and Engineering, Waseda University, Shinjuku, Tokyo 169-8050, Japan; ^5^Research Fellow for the Japan Society for the Promotion of Science, Chiyoda, Tokyo 102-0083, Japan; ^6^Section of Brain Function Information, National Institute for Physiological Sciences, Okazaki, Aichi 444-8585, Japan; ^7^Core for Spin Life Science, Okazaki Collaborative Platform, National Institute for Physiological Sciences, Okazaki, Aichi 444-8585, Japan; ^8^Physiological Science Program, The Graduate University for Advanced Studies (SOKENDAI), Hayama, Kanagawa 240-0193, Japan; ^9^Research Organization of Science and Technology, Ritsumeikan University, Kusatsu, Shiga 525-8577, Japan

**Keywords:** dopamine, medial prefrontal cortex, midbrain, motivation, motor performance

## Abstract

The motivation to approach rewards and avoid negative outcomes often guides human action. However, whether these opposing motivations for actions engage common or distinct neural pathways in humans remains unresolved. To address this, we conducted a functional magnetic resonance imaging study using a ready–set–go task with potential monetary gains (approach) and losses (avoidance) in 29 healthy individuals (18 males and 11 females). At the prefrontal level, the ventromedial prefrontal cortex and dorsomedial prefrontal cortex were preferentially activated by the anticipation of gains and losses, respectively, indicating distinct cortical representations of approach and avoidance motivations. At the subcortical level, the dorsolateral ventral midbrain (VM) was engaged during the anticipation of both gains and losses, whereas the ventromedial VM was selectively activated by gain anticipation. Crucially, dorsolateral VM activity was positively correlated with the subsequent peak grip force but not the reaction time (RT), suggesting its role in transmitting motivational signals to downstream motor circuits. At the motor cortical level, the activity of the primary motor cortex was correlated with both the grip force and RT. Together, these findings suggest a hierarchical circuit in which approach and avoidance motivations are distinctly represented in the prefrontal cortex, partially converge in the VM, and ultimately influence motor cortex activity to generate motor output. This framework provides evidence that opposing motivations engage both distinct and shared neural pathways and that their integration through subcortical–cortical interactions enables motivational signals to shape human motor action.

## Significance Statement

Human actions are energized by approaching rewards and avoiding punishments, but how these opposing motivated actions converge in the brain remains unclear. Using functional magnetic resonance imaging during a motivational ready–set–go task, we discovered that approach and avoidance motivations engage distinct prefrontal representations that partially converge in the dorsolateral ventral midbrain. This region encoded motivational intensity regardless of valence and predicted peak grip force, but not reaction time, suggesting its role in scaling motor output strength, a key component of motor vigor. These findings reveal a hierarchical circuit in which opposing motivations are differentiated cortically, integrated subcortically, and transformed into motor output through mesocortical pathways. This advances understanding of motivation‒motor interactions, with implications for disorders affecting motivated action.

## Introduction

Motivation substantially influences human action. Reward anticipation increases motor vigor, as reflected in faster responses and greater force production (Carter et al., 2009; Mir et al., 2011; Sugawara et al., 2023; Marbaker et al., 2025). Larger rewards further increase movement speed and force (Pessiglione et al., 2007; Manohar et al., 2015; Sugawara et al., 2023). Likewise, avoidance motivation—acting to prevent potential losses—shortens reaction times (RTs; Hardin et al., 2006; Carter et al., 2009), indicating that both positive and negative motivational valences energize actions. However, how the brain encodes opposing motivational valences and translates them into action remains incompletely understood.

At the cortical level, subregions of the medial prefrontal cortex (mPFC) contribute differentially to motivational processing. Activity in the ventromedial prefrontal cortex (vmPFC) is associated with reward anticipation and approach behavior (Knutson et al., 2005; Monosov and Hikosaka, 2012; Clithero and Rangel, 2014), whereas the dorsomedial prefrontal cortex (dmPFC) and dorsal anterior cingulate cortex (dACC) are preferentially engaged during anticipation of losses or punishment (Liu et al., 2011; Dugré et al., 2018). This suggests functional dissociation within the mPFC, where approach and avoidance motivations might be represented by distinct neural populations. However, how these cortical representations communicate with subcortical motivational circuits—such as the ventral midbrain (VM) and striatum—that mediate incentive salience and motor vigor remains unclear. Understanding how prefrontal value representations interface with motivational and motor circuits is essential for elucidating the neural pathways through which motivation shapes action.

Subcortical dopaminergic systems, particularly the VM, encompassing the ventral tegmental area (VTA) and substantia nigra pars compacta (SNc), are critical for linking motivational drive with motor outputs. VM dopamine neurons encode reward predictions (Schultz et al., 1997; Tobler et al., 2005; D’Ardenne et al., 2008) and motivational salience signals, which are defined as the absolute (unsigned) value of expected outcomes that increase with the magnitude of both rewards and punishments (Bromberg-Martin et al., 2010). Through widespread dopaminergic projections to the limbic, prefrontal, and motor cortices (Williams and Goldman-Rakic, 1998; Haber and Knutson, 2010), the VM is anatomically positioned to influence both motivational evaluation and movement preparation. In nonhuman primates, ventromedial and dorsolateral VM neurons exhibit reward-specific and valence-general responses, respectively, revealing a topographic organization that differentiates approach and avoidance signals (Matsumoto and Hikosaka, 2009). Such segregation implies that motivational valence may be represented by separate but interacting neural populations within the VM. Human neuroimaging studies further support the role of the VM in linking motivation to action. VM preparatory activity has been shown to predict the magnitude of forces generated during motivated action, indicating that midbrain motivational signals are directly coupled to scaling motor output strength (Sugawara et al., 2023). These findings position the VM as a central hub that transforms motivational value into action-related processes. However, whether approach and avoidance motivations engage shared or distinct VM pathways in humans and how these signals differentially influence action remain unclear.

In the present study, we combined functional magnetic resonance imaging (fMRI) measurements with a ready–set–go task adapted from the Monetary Incentive Delay (MID) task (Knutson et al., 2000), incorporating monetary gains (approach) and losses (avoidance). This design enabled systematic manipulation of motivational valence and intensity as well as a trial-by-trial quantification of two dissociable indices of motor output: RT, reflecting movement initiation, and peak grip force, reflecting movement vigor. By focusing on anticipatory ready-period brain activity, we identified cortical and subcortical mechanisms engaged prior to movement initiation. This approach provides a framework for understanding how approach and avoidance motivations are represented across hierarchical brain systems and how these motivational states transform into preparatory neural activity that shapes action.

## Materials and Methods

### Participants

Thirty healthy volunteers (19 males and 11 females; mean age ± SD = 22.13 ± 2.76 years) participated in this study. None of the participants had a history of neurological or psychiatric disorders. All participants had normal or corrected-to-normal vision and were right-handed on the basis of Edinburgh's Handedness Inventory (Oldfield, 1971). Written informed consent was obtained from all participants before participation in the experiments, and the study was conducted in accordance with the Declaration of Helsinki. This study was approved by the Ethical Committee of the National Institute for Physiological Sciences, Japan (approval number 17A02), and the Tokyo Metropolitan Institute of Medical Science, Japan (approval number 17-3). Data from one male participant were excluded from further analyses due to an abnormally high success rate in a single fMRI session, which prevented the construction of a consistent first-level design matrix across participants.

### Behavioral task

The participants lay in an MRI scanner (Verio; Siemens Healthcare) with their heads immobilized by an elastic band and sponge cushions and with their ears plugged. Stimulus presentation and response collection were conducted using the Presentation software (Neurobehavioral Systems) implemented on a personal computer (dc7900; Hewlett-Packard). An LCD projector (CP-SX12000; Hitachi) was located outside and behind the scanner. The stimuli were projected through a waveguide to a translucent screen, which the participants viewed via a mirror placed inside the MRI scanner. The spatial resolution of the projector was 1,024 × 768 pixels, with a 60 Hz refresh rate. The distance between the screen and each of the subjects' eyes was ∼175 cm, and the visual angle was 13.8° (horizontal) × 10.4° (vertical). The participants held an MRI-safe optical grip-force device (Current Designs) with their dominant right hand during the scans.

We first measured the participants' maximum grip force three times to determine the individual threshold for a participant providing a response. In this task, participants were instructed to squeeze the device as strongly as possible, while the instruction message “Squeeze” was displayed in Japanese for 5 s and to release their force while the instruction message “Rest” was displayed in Japanese for 5 s. We estimated the average maximum force power across three measurements as the maximum voluntary contraction (MVC).

Next, to determine the RT threshold, which was used to categorize each response as success or failure, the participants completed a simple ready–set–go task. This task was adapted from the MID task (Knutson et al., 2000, 2001, 2003) and modified to incorporate a ready–set–go structure that allowed trial-by-trial quantification of both RT and peak grip force. In each trial, the ready cue, represented as an open white triangle (cue stimulus), appeared for 250 ms. After this cue stimulus disappeared, a white-colored crosshair was presented for a variable interval (1,250–2,250 ms) as a delay period. Then, when a solid green square (go stimulus) was displayed for 300 ms, participants responded by squeezing the grip-force device (Current Designs) as quickly as possible. After the go stimulus disappeared, a feedback message was displayed on the screen for 1,000 ms. If a participant responded faster than the RT threshold explained below, the word “Success” was displayed. If a participant responded slower than the RT threshold, the word “Failure” was displayed. Intertrial intervals (ITIs) were varied by the duration of the delay periods (1,200–2,200 ms).

**Figure 1.**
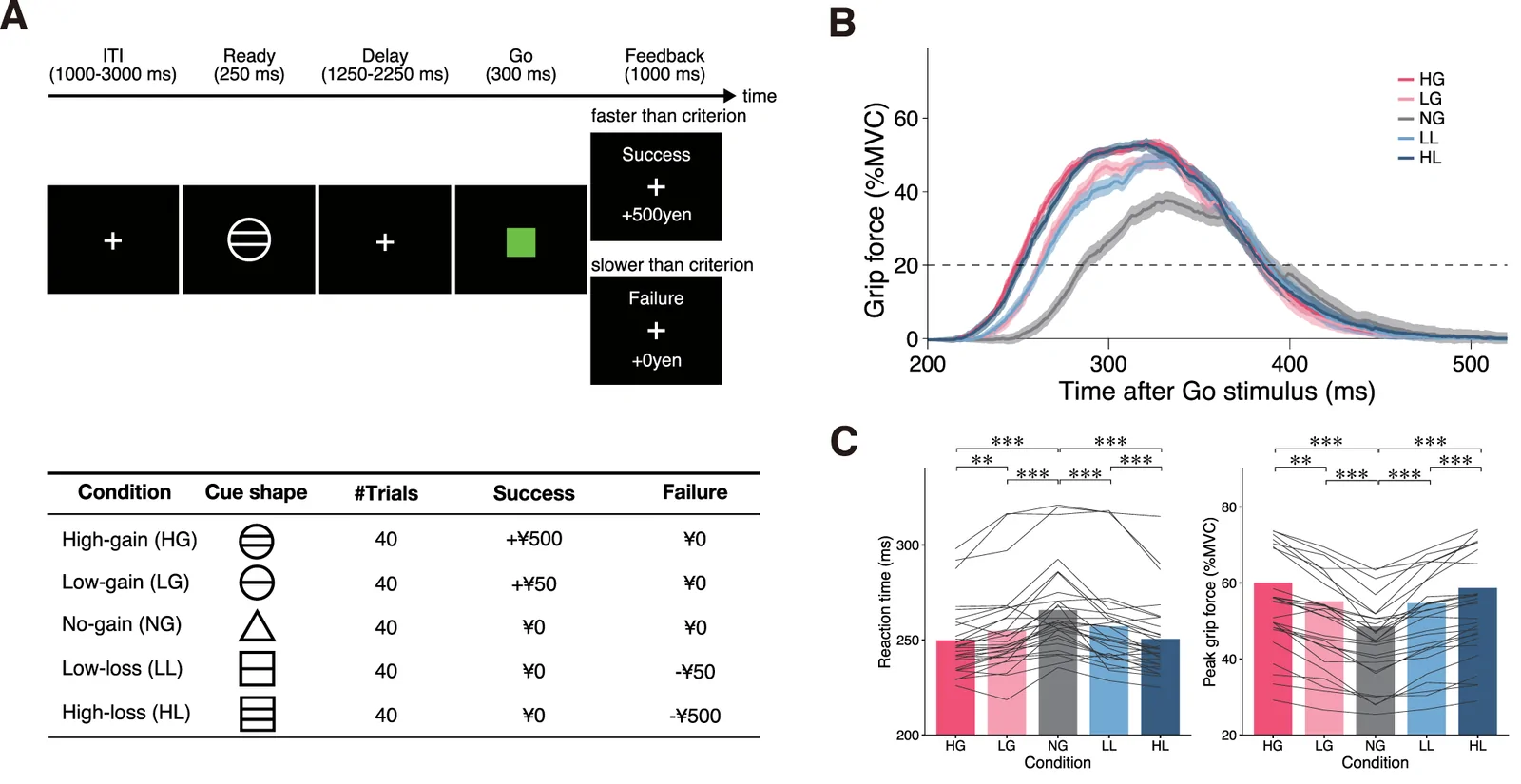
Behavioral paradigm and motor performance. ***A***, Incentive ready–set–go task. Participants were asked to prepare to respond to the cue stimulus and squeeze a grip sensor to respond to the go stimulus (green square) as quickly as possible. To manipulate motivational states, this task included five conditions corresponding to the different amounts of monetary gains for faster responses and losses for slower responses: HG (+¥500/−¥0), LG (+¥50/−¥0), NG (+¥0/−¥0), LL (+¥0/−¥50), and HL (+¥0/−¥500). ***B***, Representative grip force trajectory in each condition. Data were obtained from the subject N.M. under five conditions: HG (red), LG (light red), NG (gray), LL (light blue), and HL (blue). The lines depict the mean grip force, and the shaded areas denote the standard error of the mean (SEM). ***C***, Motor performance results (*N* = 29). The median RTs (left) significantly differed across conditions, with most pairwise comparisons showing a significant difference. Additionally, the median peak grip force differed significantly across conditions, with notable differences among almost all pairs. The bars represent the mean RTs (left panel) and peak grip forces (right panel) for each condition. The lines indicate the data of individual participants for each condition. ***p < 0.001 and **p < 0.01.

First, participants performed two short practice sessions (10 trials each) to familiarize themselves with the task and to obtain baseline RTs. The RT threshold was initially set to the median RT in the second practice block. Participants then performed one session of the simple ready–set–go task, after which the threshold was updated to the median RT obtained in this session. This single participant-specific threshold was then held fixed throughout the subsequent incentive ready–set–go task described below. The threshold was applied uniformly across trials regardless of incentive condition and was not adaptively updated between sessions or trials. Following the simple ready–set–go task, the participants performed four sessions of the incentive ready–set–go task (Fig. 1*A*). Although the trial procedure was identical to that of the simple ready–set–go task, this task had five conditions corresponding to five cues: high gain (HG), low gain (LG), no gain (NG), low loss (LL), and high loss (HL). An open white circle with two horizontal lines, open white circle with one horizontal line, open white triangle, open white square with one horizontal line, and open white square with two horizontal lines denoted the HG, LG, NG, LL, and HL conditions, respectively. In the HG and LG conditions, participants received 500 and 50 Japanese yen per trial, respectively, if their response was faster than the RT threshold. On the other hand, in the HL and LL conditions, participants lost 500 and 50 Japanese yen per trial, respectively, if their response was slower than the RT threshold. In the NG condition, participants received neither monetary gain nor loss for any response. Each session included 50 task trials (10 trials per condition) and 10 null trials in which only the fixation cross was displayed for 5 s.

### Behavioral data analysis

The grip force trajectory during each trial was recorded. On the basis of these trajectories, the RT and maximum force were estimated in a trial-by-trial manner. The RT was defined as the time at which the force reached 20% of the MVC. The maximum force was defined as the maximum force recorded after the presentation of the target stimulus.

All trials were divided into three types according to performance: correct, omission, and false start. Trials in which the grip force power did not reach 20% of the MVC from the onset of the go stimulus to the onset of feedback were defined as “omission” trials. Trials in which the grip force power reached 5% of the MVC before the onset of the go stimulus were defined as “false-start” trials. The other trials were defined as “correct” trials. Individual motor performance was calculated only for the correct trials. For individual motor performance, the median RT and peak grip force were calculated for each incentive condition. In addition, the proportion of correct trials was calculated as the correct rate. Furthermore, the numbers of omission and false-start trials in each incentive condition were calculated.

To test the effect of the incentive cue on behavioral performance, we conducted a one-way repeated–measures analysis of variance (rmANOVA) with conditions (HG/LG/NG/LL/HL) for the response time and the maximum force. Then, post hoc comparisons were performed by using a paired *t* test with Bonferroni’s correction. To quantify the trial-by-trial relationship between RT and peak grip force, we computed Kendall's rank correlation between the two motor parameters across correct trials for each participant. The correlation was calculated separately for each of the five incentive conditions (HG, LG, NG, LL, HL) and across all conditions pooled together. To test whether the group-level distribution of tau values differed from zero, we performed one-sample Wilcoxon signed-rank tests for each of the six distributions. The resulting *p* values were corrected for multiple comparisons using Bonferroni’s method. The statistical level was set to 0.05 for all the behavioral analyses.

### MRI data acquisition

A 3.0 T scanner (Verio; Siemens) was used for the fMRI study. fMRI recordings were performed using a multiband gradient-echo (GRE) echoplanar imaging (EPI) sequence [Moeller et al., 2010; multiband factor, 6; repetition time (TR), 1,000 ms; echo time (TE), 35 ms; field of view (FOV), 192 × 192 mm^2^; flip angle, 65°; matrix size, 96 × 96; 60 slices; slice thickness, 2.0 mm; phase partial Fourier, 6/8]. The number of scans was 390 per run. In this sequence, a single-band GRE-EPI image was also acquired using separate single-band excitations of the same slices. Two spin‒echo (SE) EPI images with reversed phase encoding (PE) directions were also acquired (TR, 7,560 ms; TE, 64 ms; FOV, 192 × 192 mm^2^; FA, 90/180°; matrix size, 96 × 96; 60 slices; slice thickness, 2 mm; phase partial Fourier, 6/8). These images, referred to as SE field maps, were used to correct susceptibility-induced distortions with the topup tool of FSL (FMRIB Software Library; https://fsl.fmrib.ox.ac.uk/fsl/fslwiki) using a method similar to that described by Andersson et al. (Andersson et al., 2003).

To apply the HCP pipeline to analyze our data, we scanned a series of structural images from all participants on another day. Two separate T1-weighted (T1w) and T2-weighted (T2w) images were acquired using a 3D magnetization-prepared rapid-acquisition GRE sequence (Mugler and Brookeman, 1990; TR, 2,400 ms; TE, 2.24 ms; FOV, 256 × 240 mm^2^; flip angle, 8°; matrix size, 320 × 320; slice thickness, 0.8 mm; 224 sagittal slices; GRAPPA, 2) and a variable flip angle turbo SE sequence (Siemens SPACE; Mugler et al., 2000; TR, 3,200 ms; TE, 560 ms; FOV, 256 × 240 mm^2^; matrix size, 320 × 320; slice thickness, 0.8 mm; 224 sagittal slices; GRAPPA, 2), respectively. Similar to the functional MRI session, two SE-EPI sequences with reversed PE directions were acquired (TR, 7,700; TE, 60 ms; FOV, 192 × 192 mm^2^; FA, 78/160°; matrix size, 98 × 98; 72 slices; slice thickness, 2 mm; phase partial Fourier, 6/8).

### MRI data preprocessing

All MRI data were preprocessed using HCP minimum preprocessing pipelines (Glasser et al., 2013; Yamamoto et al., 2021, 2025). Structural images were preprocessed according to the following processes: gradient magnetic field nonlinearity-induced distortion correction (GDC) with the gradient coefficient file of the scanner, image averaging in each T1w and T2w image for signal-to-noise ratio (SNR) improvement after motion correction, susceptibility-induced distortion correction (SDC) in the frequency encoding (superior-to-inferior) direction with the SE field maps, cross-modal registration between the averaged T1w and T2w images using the boundary-based registration (Greve and Fischl, 2009), and bias field correction with the square root of the multiplication of T1w and T2w images. The preprocessed structural images in the participants' native space were nonlinearly normalized to the Montreal Neurological Institute (MNI) space. The task fMRI data were preprocessed as follows: GDC, motion correction to align to the single-band GRE-EPI image in each run, SDC in the PE (AP or PA) direction, registration to the preprocessed T1w image via the single-band GRE-EPI image, bias field correction using the single-band GRE- and SE-EPI images, nonlinear image normalization to the MNI space, intensity normalization, and spatial smoothing with a Gaussian kernel of 5 mm FWHM.

### MRI data analysis

After HCP-style preprocessing, we analyzed task-related activity with the Statistical Parametric Mapping software (SPM12; The Wellcome Trust Centre for Neuroimaging) in MATLAB 2018a. The first five volumes of each fMRI run were discarded because the signal was unsteady. Statistical analysis of the fMRI data was conducted at two levels. At the first level, a general linear model (GLM) was fitted to the fMRI data for each participant (Friston et al., 1994; Worsley and Friston, 1995).

Three types of GLMs were applied to identify the brain regions associated with the effects of incentive conditions and subsequent motor performance during the ready phase. To illustrate the brain regions associated with the effect of incentive conditions on preparatory activity, GLM1 modeled the five experimental conditions (HG, LG, NG, LL, and HL) for the ready cue as separate regressors. Additionally, the go and feedback periods were modeled as the same epoch with a duration of 1,300 ms. These epochs were divided into four regressors corresponding to success/failure and gain/loss factors: success with gain (SG), success without gain (SN), failure without loss (FN), and failure with loss (FL). This four-way partitioning classified each trial according to its outcome and monetary consequence, ensuring that outcome-related variance was modeled during the go/feedback epoch rather than being attributed to the anticipatory ready cue regressors of interest. Thus, GLM1 contained nine regressors: five for the three incentive conditions in the ready cue display, modeled with a stick function, and four for the go and feedback epoch, modeled with a boxcar function (1,300 ms fixed duration). GLM2 contained two regressors, modeling the events of a trial with a stick function for the ready cue and a boxcar function (1,300 ms fixed duration) for the go and feedback epoch. To illustrate the brain regions related to subsequent motor performance exerted in response to the go display, the ready-related regressor was parametrically modulated by the RT and peak grip force. GLM3 applied the same model as GLM2 to data from the simple ready–set–go task performed by the same participants before the incentive ready–set–go task. Because this task did not involve monetary incentives, it provided a dataset independent of the main incentive task analyses, which was used to define regions of interest (ROIs) showing premovement activity without circular inference.

For all the GLMs, all the regressors were convolved with the canonical hemodynamic response function implemented in SPM12, which is modeled as a mixture of two gamma functions representing the response peak (∼6 s poststimulus) and the subsequent undershoot (∼16 s poststimulus; Friston et al., 1998). Additionally, to consider the effect of head motion, 24 nuisance regressors modeling head motions (derived from six motion parameters, their first temporal derivatives, and the square of these 12 resulting regressors) were included in each GLM (Satterthwaite et al., 2013). Two additional regressors, describing intensities in cerebrospinal fluid and white matter compartments, were added to the models. The time series for each voxel was high-pass filtered at 1/128 Hz. With respect to the advanced rapid sampling techniques such as multiband EPI used in this study, a first-order autoregressive model does not sufficiently capture temporal correlations in time series data with higher sampling rates (Bollmann et al., 2018). Thus, the “FAST” model implemented in SPM12 was applied to adequately address the temporal correlations in time series data with higher sampling rates (Corbin et al., 2018). Then, to calculate the estimated parameters, a least-squares estimation was performed on the high-pass filtered and prewhitened data.

The parameter estimates for each regressor of interest in each model were subjected to second-level analysis (Holmes and Friston, 1998) with a flexible–factorial model. In GLM1, eight linear contrasts were estimated to characterize anticipatory activity related to monetary incentives. To identify brain regions associated with the anticipation of monetary gains, we computed two contrasts: the gain-related contrast ([HG + LG] − NG), capturing activation linked to the prospect of monetary gains relative to the no-incentive baseline, and the gain-magnitude contrast (HG − LG), capturing activation scaling with the amount of potential gains. Similarly, to identify brain regions associated with the anticipation of monetary losses, we computed two contrasts: the loss-related contrast ([HL + LL] − NG) and the loss-magnitude contrast (HL − LL). Moreover, to identify brain regions commonly engaged across gain and loss contexts, we performed two conjunction analyses using a logical AND. The common contrast ([HG + LG] − NG ∩ [HL + LL] − NG) identified regions activated by the anticipation of both gains and losses relative to the no-incentive baseline. The motivational salience contrast ([HG − LG] ∩ [HL − LL]) identified regions whose activity scaled with the magnitude of monetary incentives regardless of valence, corresponding to an unsigned value signal (Kahnt and Tobler, 2017). Finally, to identify brain regions selectively engaged by motivational valence, we further computed the gain-specific contrast ([HG + LG] − [HL + LL]) and the loss-specific contrast ([HL + LL] − [HG + LG]).

In GLM2, two contrasts were estimated. The parametric modulations with the subsequent RTs and peak grip forces were calculated. To identify brain regions jointly modulated by both motor parameters, a conjunction analysis using a logical AND was performed on the two parametric modulation contrasts. Furthermore, to directly compare the neural representations of the two motor parameters, the corresponding first-level parametric modulation maps were entered into a second-level flexible–factorial model with modulator type as a within-participant factor, with two levels (RT and peak grip force). Two contrasts related to differences in parametric modulations were then estimated: (RT modulation) > (peak grip force modulation) and (peak grip force modulation) > (RT modulation). The resulting set of voxel values for each contrast constituted SPM{t}, which was transformed into normal distribution units (SPM{z}).

For the whole-brain inference, statistical significance was assessed at the cluster level using familywise error (FWE) correction at *p* < 0.05, with a cluster-forming voxel-level threshold of *p* < 0.001 uncorrected (corresponding to *Z* = 3.09), based on Gaussian random field theory (Friston et al., 1996). Typically, the MRI signals in the ventral region of the brain, including the nucleus accumbens (NAc) and midbrain, are weaker than the signals in lateral cortical regions. Thus, the automatically defined brain masks created in group-level analyses often lack ventral brain regions. To address this issue, we used the explicit brain mask made by averaging the extracted individual brain masks created via the structural pipelines. Moreover, we conducted a small-volume correction analysis for all the GLMs to precisely evaluate the activities in the VM region. Subcortical volumes of interest, including those of the VM, NAc, and caudate nucleus, were defined based on the probabilistic MRI-based atlas of human subcortical brain nuclei (Pauli et al., 2018). The VM was defined as the midbrain region including the SNc, the parabrachial pigmented nucleus (PBP), and the VTA. The same atlas was used for small-volume correction over the VM mask. The statistical threshold for small-volume correction was set at *p* < 0.05 and corrected for multiple comparisons over the VM mask. Additionally, the extent threshold for the VM mask was set as 5 voxels.

Brain regions were automatically defined and labeled according to an automated anatomical labeling atlas (Tzourio-Mazoyer et al., 2002; Rolls et al., 2020), the “Human Motor Area Template” (Mayka et al., 2006), and a probabilistic in vivo atlas of human subcortical nuclei for subcortical structures, including the midbrain, NAc, and caudate nucleus (Pauli et al., 2018).

To visualize the activity profile across the five conditions within significant clusters, we extracted mean beta estimates from each significant cluster of the common, motivational salience, gain-specific, and loss-specific contrasts. For the gain-specific contrast, the largest cluster spanned both the vmPFC and the NAc. To separately characterize the activity in these functionally distinct nodes of the reward circuit, we additionally defined anatomical sub-ROIs and intersected them with the cluster mask. The vmPFC sub-ROIs (left and right separately) were defined as the union of Areas 32d, 24, 14m, and 25 from the Cingulate and Orbitofrontal Parcellation atlas (Neubert et al., 2015). The NAc sub-ROIs (left and right separately) were defined from a probabilistic in vivo atlas of human subcortical nuclei (Pauli et al., 2018). Mean beta estimates within each extraction mask were computed for each participant and each of the five incentive conditions.

## Results

### Motor performance

We measured the grip force to quantify how motivational states influence motor performance (*N* = 29 participants). The averaged grip force trajectories for each condition for a representative participant are shown in Figure 1*B*. Grip force trajectories were strongly influenced by the expected monetary outcome (Fig. 1*B*). At the group level, the peak grip force and RT varied significantly across conditions (Fig. 1*C*; peak grip force, one-way rmANOVA, *F*_(1.49,41.76)_ = 44.01; *p* = 1.14 × 10^−9^; RT, one-way rmANOVA, *F*_(2.84,79.53)_ = 31.24; *p* = 4.71 × 10^−13^). Regardless of whether the context was gain or loss, different expected outcomes resulted in significant differences in both the peak grip force and RT (Bonferroni’s corrected pairwise comparisons; peak grip force, HG vs LG, *p* = 2.34 × 10^−7^; HG vs NG, *p* = 2.34 × 10^−7^; HG vs LL, *p* = 7.81 × 10^−6^; LG vs NG, *p* = 3.12 × 10^−5^; LG vs HL, *p* = 1.12 × 10^−5^; NG vs LL, *p* = 3.69 × 10^−7^; NG vs HL, *p* = 3.89 × 10^−7^; LL vs HL, *p* = 2.01 × 10^−5^; RT: HG vs LG, *p* = 3.65 × 10^−3^; HG vs NG, *p* = 2.25 × 10^−7^; HG vs LL, *p* = 1.31 × 10^−4^; LG vs NG, *p* = 1.55 × 10^−5^; NG vs LL, *p* = 2.97 × 10^−4^; NG vs HL, *p* = 1.29 × 10^−7^; and LL vs HL, *p* = 4.24 × 10^−3^). Peak grip force was greater in HG than in HL by 1.38 ± 0.36% of the MVC (*p* = 1.34 × 10^−3^). Otherwise, neither peak grip force nor RT differed significantly between conditions with the same amount of expected gain and loss (peak grip force, LG vs LL, *p* = 0.46; RT, HG vs HL, *p* = 0.57; LG vs LL, *p* = 0.089). These findings suggest that the effect of incentive motivation on motor performance cannot be fully explained by motivational context alone.

### Whole-brain activity related to anticipating monetary gains and losses

To investigate the neural substrates that mediate approach and avoidance motivations, we examined premovement activity during the anticipatory ready phase across the entire brain (cluster-level FWE–corrected *p* < 0.05 with peak-level uncorrected *p* < 0.001). Both the RT and grip force improved proportionally with the degree of incentive motivation (Fig. 1*C*). Therefore, we first identified brain regions showing shared activity between gain and loss contexts using conjunction analysis ([HG + LG] > NG ∩ [HL + LL] > NG).

**Figure 2.**
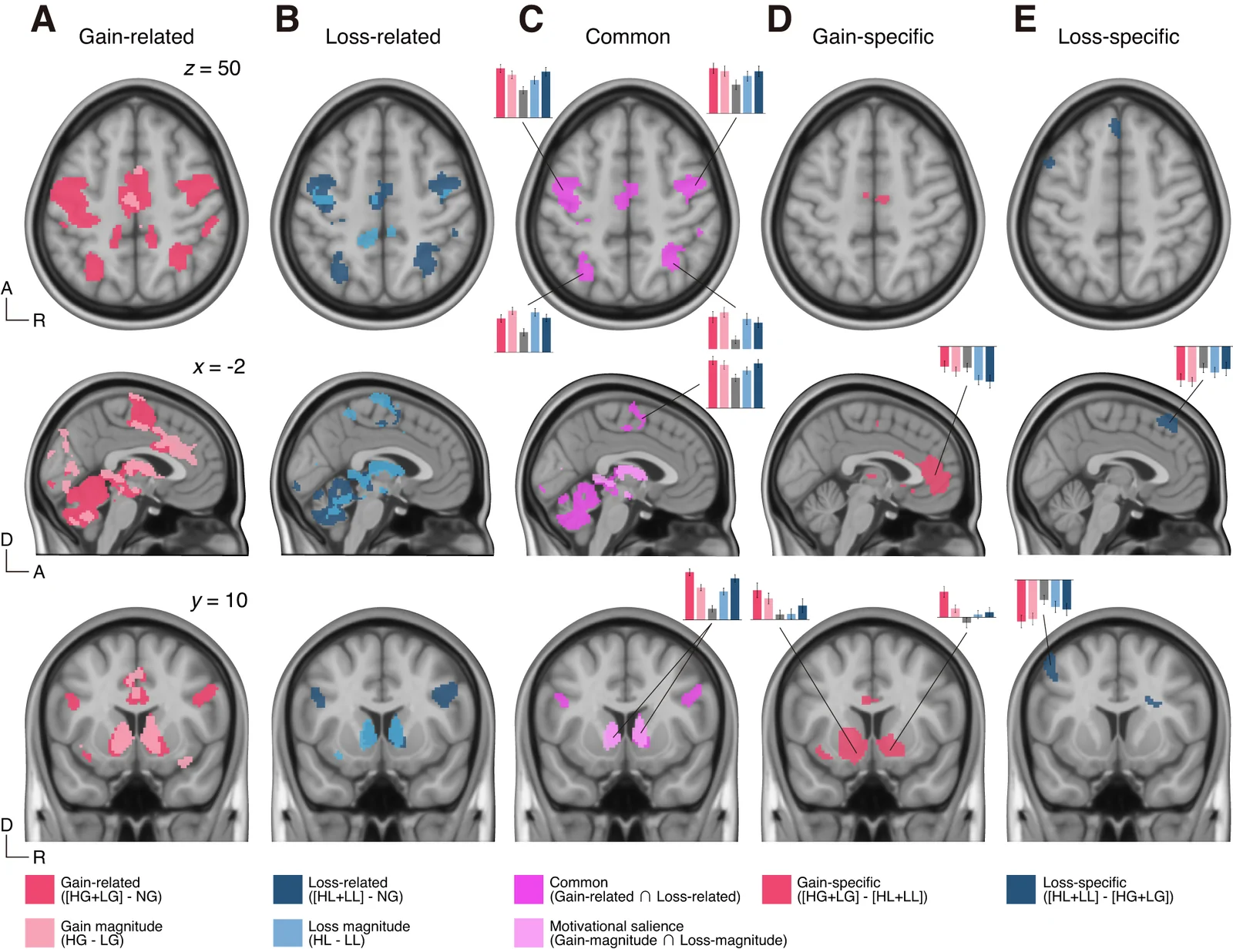
Corticostriatal premovement activities related to anticipating monetary gains and losses. ***A***, The brain activities related to monetary gains (gain-related: [HG + LG] > NG) and the magnitude of potential gains (gain-magnitude: HG − LG). ***B***, The brain activities related to monetary losses (loss-related: [HL + LL] > NG) and the magnitude of potential losses (loss-magnitude: HL − LL). ***C***, Common activities between gain and loss conditions (common: [HG + LG] > NG ∩ [HL + LL] > NG) and brain activities reflecting motivational salience, defined as the unsigned value of upcoming incentives (motivational salience: [HG − LG] ∩ [HL − LL]). ***D***, Gain-specific activities ([HG + LG] > [HL + LL]). ***E***, Loss-specific activities ([HL + LL] > [HG + LG]). Bar plot insets in ***C***–***E*** show mean beta estimates extracted from significant clusters across the five conditions (HG, LG, NG, LL, HL). In ***D***, insets are additionally shown for anatomical sub-ROIs (vmPFC and NAc, left and right separately) intersected with the gain-specific clusters. Error bars represent the standard error of the mean. A, anterior; D, dorsal; R, right.

Conjunction analysis revealed widespread overlap between gain- and loss-related anticipation. At the cortical level, shared premovement activity was observed in the contralateral primary motor cortex (M1), bilateral dorsal premotor cortex (PMd), supplementary motor area (SMA), superior parietal lobule (SPL), and anterior insular cortex (Fig. 2*A*–*C*, top panels; Table S1). At the subcortical level, the bilateral caudate nucleus, bilateral thalamus, and cerebellar vermis were commonly recruited in both gain and loss contexts (Fig. 2*A*–*C*, middle and bottom panels). These regions likely constitute a core network that supports motivation-dependent motor preparation that operates independently of whether the anticipated outcome is positive or negative.

To identify brain regions encoding motivational salience independent of gain and loss contexts, we performed an additional conjunction analysis ([HG − LG] ∩ [HL − LL]). Regardless of the context, higher incentive magnitudes were associated with increased activity in the bilateral anterior insular cortex, caudate nucleus, and thalamus (Fig. 2*C*, middle and bottom panels), suggesting that these regions encode the magnitude of motivational drive rather than its valence.

We next tested for valence-specific preparatory activity by directly comparing the gain and loss conditions ([HG + LG] − [HL + LL] and [HL + LL] − [HG + LG]). The anticipation of potential gains and losses engaged distinct regions within the mPFC (Fig. 2*D*–*E*, top panels; Table S1). Specifically, gain-specific and loss-specific premovement activities were observed in the vmPFC (Fig. 2*D*, top panel) and dmPFC (Fig. 2*E*, top panel), respectively. Loss-specific activity was also observed in the left dorsolateral prefrontal cortex (Fig. 2*E*, top panel). In the striatum, gain-specific activity was observed in the NAc (Fig. 2*D*, bottom panel), consistent with the canonical NAc response in previous MID studies (Knutson et al., 2001; Wu et al., 2014), whereas no loss-specific activity was detected (Fig. 2*E*, bottom panel). These results indicate that approach and avoidance motivations recruit both shared and distinct preparatory networks, with the mPFC and striatum differentially representing motivational valence prior to movement initiation.

### VM activity related to the anticipation of gains and losses

Previous work on nonhuman primates has demonstrated that dopamine neurons encoding reward anticipation are concentrated in the ventromedial part of the VM, whereas those responding to both reward and punishment are distributed in the dorsolateral part, revealing a valence-based topography (Matsumoto and Hikosaka, 2009). To test whether such a similar topographic organization exists in the human brain, we examined the effect of anticipation of monetary gains and losses on premovement VM activity.

**Figure 3.**
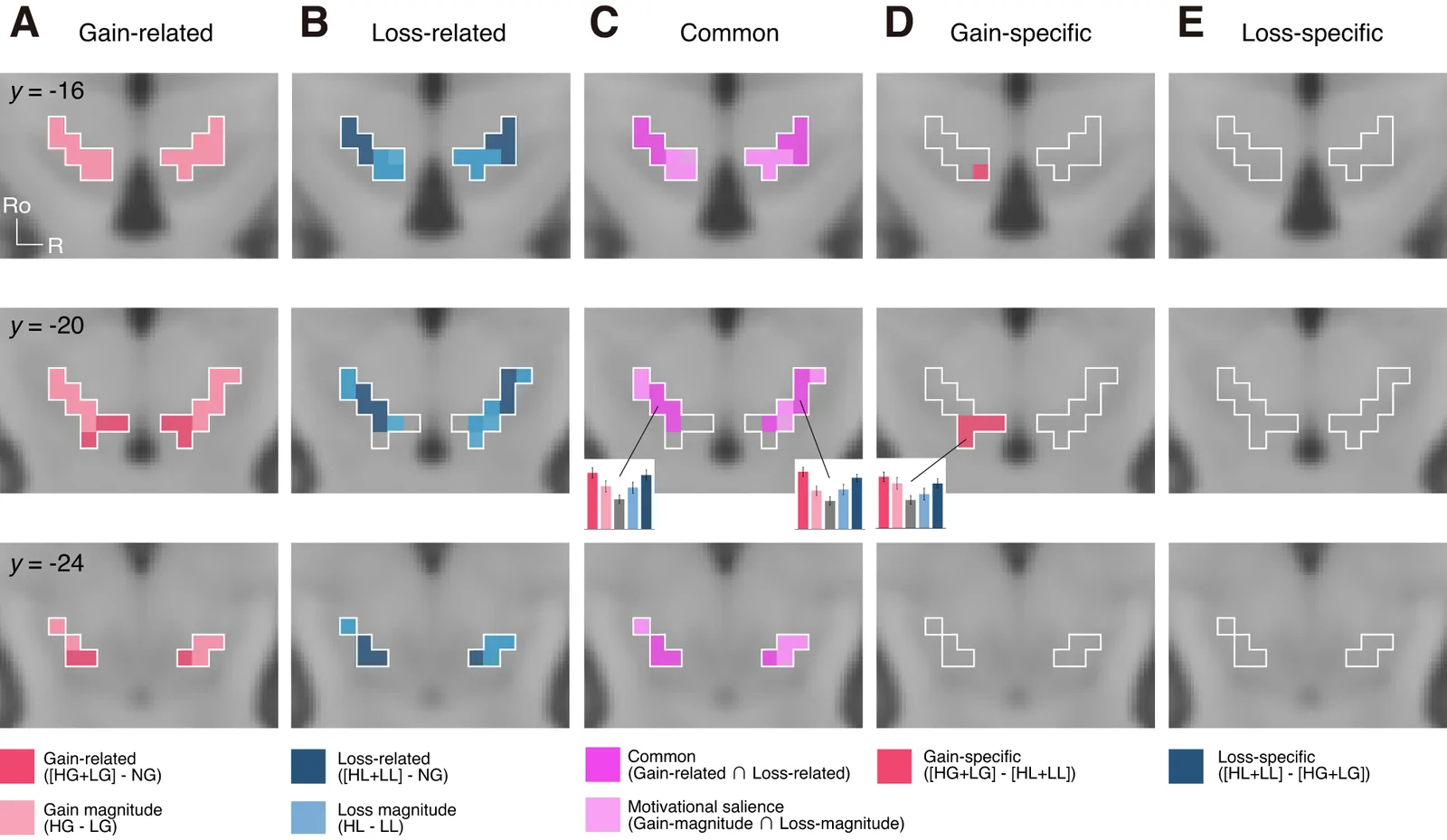
Spatial distribution of premovement activity in the VM related to anticipating monetary rewards and losses. ***A***, The VM activities related to monetary gains (gain-related: [HG + LG] > NG) and the magnitude of potential gains (gain-magnitude: HG − LG). ***B***, The VM activities related to monetary losses (loss-related: [HL + LL] > NG) and the magnitude of potential losses (loss-magnitude: HL − LL). ***C***, Common activities between gain and loss conditions (Common: [HG + LG] > NG ∩ [HL + LL] > NG) and VM activities reflecting motivational salience, defined as the unsigned value of upcoming incentives (motivational salience: [HG− LG] ∩ [HL − LL]). ***D***, Gain-specific activities ([HG + LG] > [HL + LL]). ***E***, Loss-specific activities ([HL + LL] > [HG + LG]). All panels show the coronal sections of the VM. The ROI, delineated by the white lines, encompasses the VM, including the VTA, PBP, and SNc, bilaterally. Bar plot insets in ***C*** and ***D*** show mean beta estimates extracted from significant clusters across the five conditions (HG, LG, NG, LL, HL). R, right; Ro, rostral.

Consistent with previous findings (Sugawara et al., 2023), anticipating monetary gains ([HG + LG] − NG) elicited significant activation within the VM during the premovement ready period (*p*_SVC_ < 0.05; Fig. 3*A*; Table S2). Similarly, anticipating monetary losses ([HL + LL] − NG) was associated with the activation of the VM during this period (*p*_SVC_ < 0.05; Fig. 3*B*; Table S2). Conjunction analysis revealed that a common cluster in the dorsolateral part of the VM exhibited shared premovement activity across both contexts ([HG + LG] > NG ∩ [HL + LL] > NG; *p*_SVC_ < 0.05; Fig. 3*C*; Table S2). Furthermore, a portion of this shared activity was modulated by motivational salience ([HG − LG] ∩ [HL − LL]; *p*_SVC_ < 0.05; Fig. 3*C*; Table S2), with greater VM activity in high-incentive conditions (HG and HL) than in low-incentive conditions (LG and LL). These results indicate that the dorsolateral VM encodes motivational salience independent of valence, which is consistent with its proposed role in eliciting general motivation.

To clarify whether distinct subregions within the VM are associated with approach and avoidance contexts, we calculated linear contrasts by subtracting loss from gain ([HG + LG] − [HL + LL]) and vice versa ([HL + LL] − [HG + LG]). Gain-specific VM activation was localized to the ventromedial region (*p* < 0.05; Fig. 3*D*), whereas no loss-specific activation was detected within the VM (*p*_SVC_ < 0.05; Fig. 3*E*; Table S2). This ventromedial bias for reward anticipation mirrors the topographic organization previously observed in nonhuman primates (Matsumoto and Hikosaka, 2009), suggesting that motivational intensity across contexts is integrated in the dorsolateral portion of the human VM, whereas reward specificity is associated with activity in the ventromedial region. Together, these findings provide evidence that a functionally homologous topography of general approach and valence motivational signals exists in the human VM.

### Performance-related modulations in premovement activities

**Figure 4.**
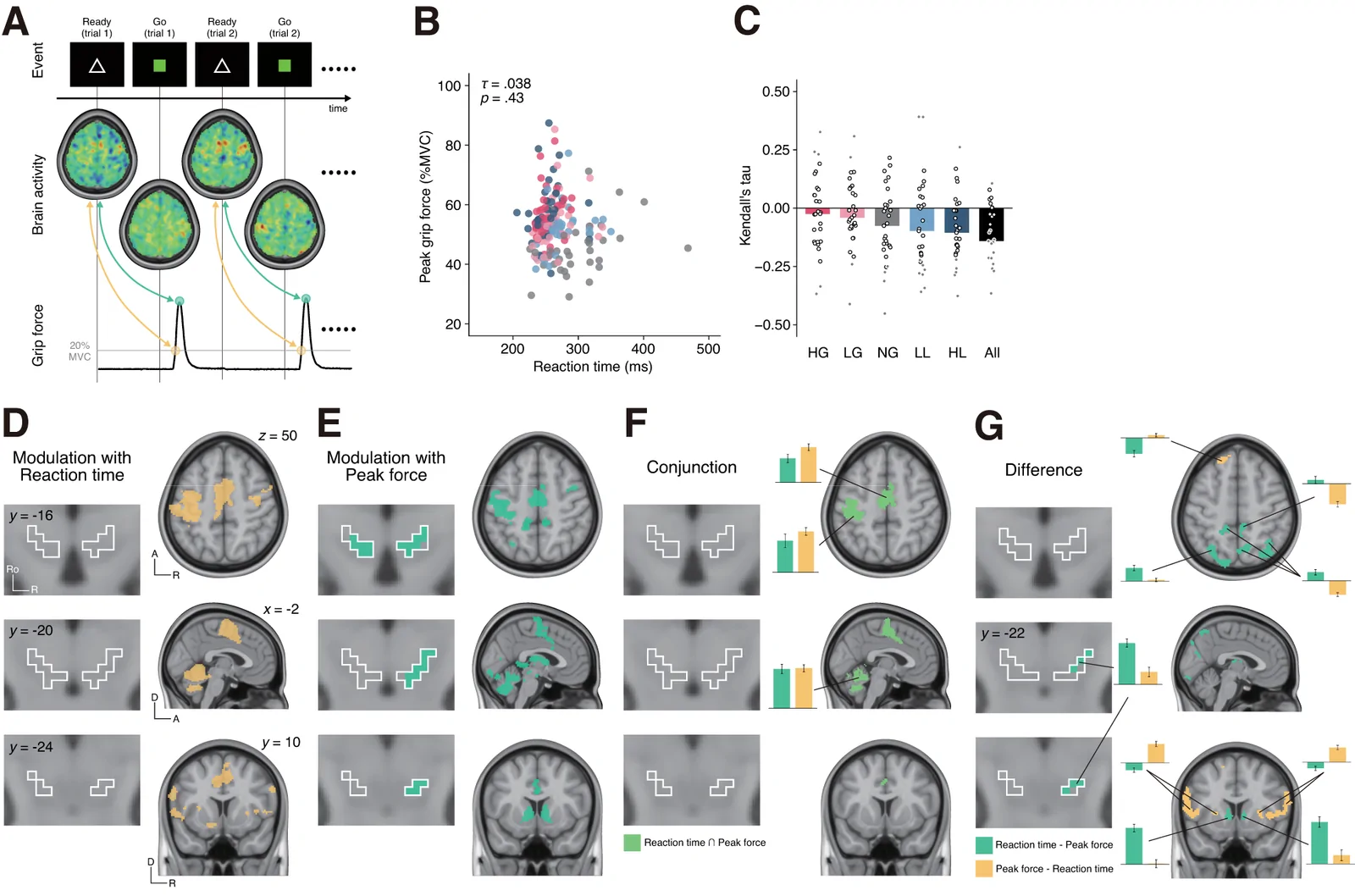
Trial-by-trial behavioral correlation and performance-related premovement activity. ***A***, Schematic of the parametric modulation analysis. To illustrate the brain regions where preparatory activity was correlated with subsequent motor performance, the contrasts modulated by the subsequent RT (yellow dots) and peak grip force (green dots) were estimated at the timing of the cue stimulus. ***B***, Trial-by-trial scatter plot of RT and peak grip force from a representative participant. Each dot represents a single trial, color-coded by condition (red, HG; light red, LG; gray, NG; light blue, LL; and blue, HL). ***C***, Distribution of within-participant Kendall's tau values between RT and peak grip force across the five incentive conditions and pooled across all conditions (black, all). Each circle represents one participant. The color of the circles indicates significant (gray) and nonsignificant (white) correlations. Bars indicate the group-level median. ***D***, Premovement activity correlated with the subsequent RT. ***E***, Premovement activity correlated with the subsequent peak grip force. ***F***, The conjunction of the two parametric modulation contrasts using a logical AND. Light green voxels indicate regions where premovement activity was significantly modulated by both RT and peak grip force. ***G***, Direct comparison of the two parametric modulation contrasts using a flexible–factorial design. Green clusters indicate voxels with significantly stronger modulation by peak grip force than by RT; yellow clusters indicate the opposite. Bar plots show contrast estimates at representative peaks. A, anterior; D, dorsal; R, right; Ro, rostral.

Previously, we demonstrated that VM premovement activity linked to reward prediction is correlated with the strength of the subsequent grip force but not with movement initiation (Sugawara et al., 2023). To replicate and extend these findings, we first confirmed the trial-by-trial independence between RT and peak grip force (Fig. 4*B*,*C*). The group-level distributions of within-participant Kendall's tau were weakly negative across all conditions (HG, median = −0.025; *V* = 193; *p* > 0.99; LG, median = −0.041; *V* = 185; *p* > 0.99; NG, median = −0.076; *V* = 104; *p* = 0.15; LL, median = −0.098; *V* = 121; *p* = 0.22; HL, median = −0.11; *V* = 86; *p* = 0.021; All, median = −0.14; *V* = 44; *p* = 3.2 × 10^−4^; one-sample Wilcoxon signed-rank tests with Bonferroni’s correction). Although the pooled distribution reached statistical significance, the magnitude of the median correlation was small, indicating that the trial-by-trial coupling between RT and peak grip force is weak. This pattern is consistent with our previous report using a gain-only ready–set–go task (Sugawara et al., 2023; median = −0.15) and suggests that the two motor parameters are at least partially controlled by distinct neural substrates.

To test this possibility at the neural level, we examined how premovement activity across cortical and subcortical regions was parametrically modulated by the subsequent RT and peak grip force (Fig. 4*A*; Tables S3, S4). In support of our previous findings, VM premovement activity was associated with the subsequent peak grip force but not with the RT (*p*_SVC_ < 0.05; Fig. 4*D*–*E*, left panels; Table S4), reinforcing its selective association with scaling motor output strength rather than action initiation. Beyond the VM, we identified a distributed set of regions in which premovement activity predicted different aspects of motor performance (cluster-level FWE–corrected *p* < 0.05 with peak-level uncorrected *p* < 0.001). During the ready period, the contralateral M1, bilateral PMd, SMA, and cerebellum exhibited premovement activity that was negatively correlated with the subsequent RT and positively correlated with the subsequent peak grip force (Fig. 4*D*–*E*, right panels; Table S3). Moreover, a conjunction analysis of the two parametric modulation contrasts confirmed that these regions were jointly associated with both motor parameters (Fig. 4*F*, left panel; Table S3). These patterns suggest that cortical and cerebellar motor-related regions collectively contribute to the timing and strength of upcoming movements. Within the striatum, premovement activity showed a dissociable relationship with RT and peak grip force. Premovement activity in the bilateral putamen correlated negatively with the subsequent RT, whereas premovement activity in the bilateral caudate nucleus correlated positively with the subsequent peak grip force (Fig. 4*D*–*E*, right bottom panels; Table S3). This functional dissociation within the striatum mirrors that observed in the VM and suggests that distinct striatal circuits contribute to movement initiation and force scaling.

Furthermore, to test whether the observed regional preferences for RT and peak grip force were statistically dissociable neural representations rather than subthreshold variation in a single shared signal, we directly compared the two parametric modulation contrasts (Fig. 4*G*; Tables S3, S4). At the whole-brain level, the bilateral putamen, bilateral ventral premotor cortex, and lateral prefrontal regions exhibited significantly stronger modulation by subsequent RT than by peak grip force (cluster-level FWE–corrected *p* < 0.05 with peak-level uncorrected *p* < 0.001; Fig. 4*G*, right panels; Table S3). Conversely, the bilateral caudate nucleus, contralateral M1 and somatosensory cortices, SMA, precuneus, and SPL showed significantly stronger modulation by subsequent peak grip force than by RT (Fig. 4*G*, right panels; Table S3). Importantly, within the VM, the right dorsolateral region exhibited significantly stronger modulation by peak grip force than by RT (*p*_SVC_ < 0.05; Fig. 4*G*, left panel; Table S4), whereas no voxel within the VM showed the opposite pattern. Taken together, these results indicate that while cortical and cerebellar networks broadly govern the initiation and scaling of voluntary movement, the striatum contributes in a more differentiated manner: the putamen facilitates rapid initiation, whereas the caudate enhances the force-generating component of action.

### Cortico–subcortical representations of subsequent performance

**Figure 5.**
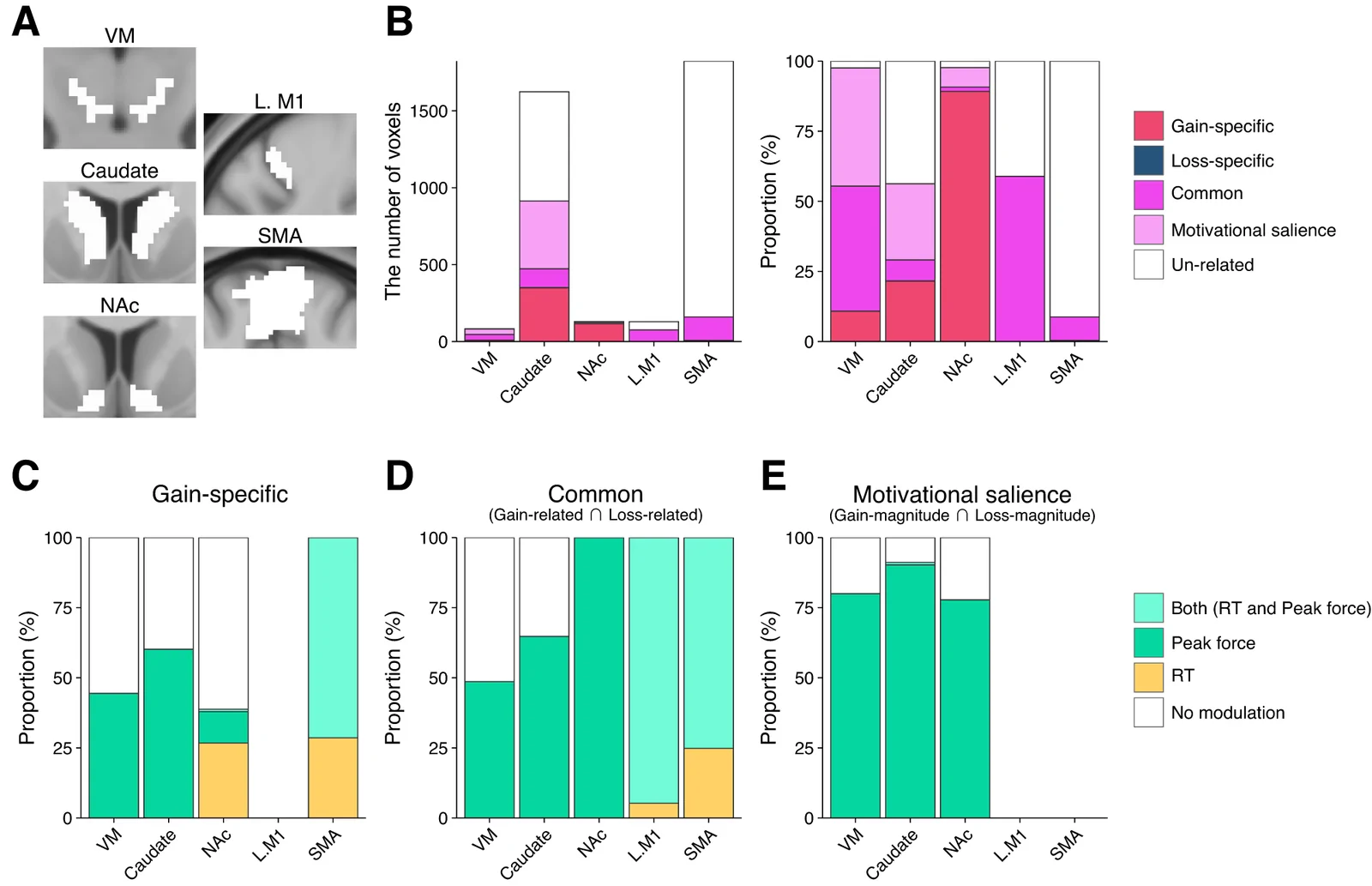
Proportion of performance-related voxels in motivation-related activations. ***A***, The ROIs included the VM, caudate nucleus, NAc, left primary motor cortex (L. M1), and bilateral SMA. ***B***, Motivation-related voxels. The left panel shows the number of voxels, whereas the right panel represents the proportion of four types of motivation-related voxels in each ROI. ***C****–**E***, Performance-related voxels. Proportion of voxels related to subsequent RT and peak grip force within (***C***) the gain-specific voxels, (***D***) the context-general common voxels, and (***E***) the salience-associated voxels.

To understand how motivation-related cortical and subcortical regions represent subsequent motor performance, we focused on five ROIs representing the core motivation–motor integration circuit. These regions were chosen on the basis of three criteria: (1) significant activation in our analyses (Figs. 2, 3), (2) established roles in the cortico–basal ganglia–midbrain circuit, and (3) representation of different hierarchical levels of motivation–motor processing. Specifically, we examined the VM as the source of dopaminergic motivational signals, the NAc, and caudate nucleus as striatal regions associated with reward processing and action–value integration, respectively, and the left M1 (contralateral to the performing hand) and bilateral SMA as cortical regions associated with motor execution and planning (Fig. 5*A*). Overall, the subcortical regions exhibited a mixture of context-general and context-specific voxel populations, whereas the cortical motor-related regions exhibited predominantly context-specific voxels with little valence selectivity (Fig. 5*B*). Within the VM, 44.6% of voxels were context-general, 42.2% were salience-associated, 10.8% were gain-specific, and none were loss-specific. The caudate nucleus showed comparable proportions of gain-specific (21.5%) and salience-associated (27.2%) voxels, with very few loss-specific (0.1%) and context-general (7.5%) voxels. The NAc was predominated by gain-specific voxels (89.2%), accompanied by a small fraction of loss-specific (1.5%), context-general (6.9%), and salience-associated (2.3%) voxels. In contrast, M1 contained only context-general voxels (58.9%). In the SMA, a small proportion of voxels were gain-specific (0.4%) and context-general (8.4%), with no loss-specific or salience-associated voxels. These distributions indicate that motivation-related signals are represented more heterogeneously in subcortical regions than in cortical motor areas, with the VM and caudate encoding both motivational salience and gain-specific signals, whereas motor cortical regions primarily reflect context-independent preparatory activity.

To determine whether these motivation-related voxel populations are linked to subsequent motor performance, we quantified the proportion of voxels whose premovement activity correlated with the subsequent RT and peak grip force within each subregion. In particular, gain-specific, context-general, and salience-associated voxels in the five ROIs were examined (Fig. 5*C*–*E*). Among gain-specific voxels (Fig. 5*C*), 44.4% (4/9) in the VM and 60.2% (210/349) in the caudate nucleus were correlated exclusively with the subsequent peak grip force. In the NAc, 11.2% (13/116) were correlated with only the peak grip force, whereas 26.7% (31/116) were correlated with only the RT. In the SMA, 28.6% (2/7) of the voxels were correlated with only the RT, whereas 71.4% (5/7) were correlated with both the RT and peak grip force. For context-general voxels (Fig. 5*D*), distinct patterns emerged between motivation-related subcortical and motor-related cortical regions. In the VM (48.6%; 18/37), caudate (64.8%; 79/122), and NAc (100%; 2/2), correlations were observed exclusively with the peak grip force, suggesting a selective association with motor output strength, which is a component of motor vigor. Conversely, in motor-related cortical regions such as M1 and the SMA, 75.2–94.7% of voxels (M,: 72/76 [94.7%]; SMA, 115/153[75.2%]) were correlated with both the RT and grip force, while smaller proportions of voxels (M1, 4/76 [5.3%]; SMA, 38/153 [24.8%]) were correlated with only the RT. Salience-associated voxels were confined to subcortical regions (Fig. 5*B*). Within these areas, the voxels in the VM (80.0%; 28/35), caudate (90.3%; 398/441), and NAc (77.8%; 7/9) were correlated solely with the peak grip force (Fig. 5*E*), indicating a strong association between force generation and motivational salience processing. Together, these results demonstrate region-specific processing in the integration of motivation and motor control, with motivation-related subcortical regions primarily controlling the peak grip force, whereas motor-related cortical regions control both the RT and peak grip force.

## Discussion

Using functional neuroimaging with an incentive ready–set–go task (a variant of the MID task) that manipulated both motivational valence and incentive magnitude, we identified hierarchical brain networks that both differentiated and integrated these opposing motivational states to shape human action. Motivational valence is dissociated at the prefrontal level, partially integrated within the VM, and translated into motor output. Notably, dorsolateral VM activity predicted subsequent force production but not movement initiation, indicating a selective role in transmitting motivational drive.

### Distinct prefrontal representations of motivational valence

At the cortical level, we observed clear functional dissociation within the mPFC: the vmPFC was preferentially activated during gain anticipation (Fig. 2*D*), whereas the dmPFC showed stronger responses to loss anticipation (Fig. 2*E*). This aligns with previous work indicating that the vmPFC is involved in reward-related decision-making and approach behavior (Knutson et al., 2005; Clithero and Rangel, 2014) and that the dmPFC is involved in negative outcomes processing, conflict monitoring, and loss-related behavioral adjustment (Liu et al., 2011; Dugré et al., 2018). Anatomically, the vmPFC receives dopaminergic projections predominantly from the medial VM, including the PBP and linear nuclei (Williams and Goldman-Rakic, 1998), and is well positioned to integrate reward prediction with outcome valuation through its connections with the NAc (Haber and Knutson, 2010).

Importantly, activity in these prefrontal regions did not correlate with the subsequent RT or grip force. Instead, only activity in the premotor cortex and M1 did (Fig. 4*D*,*E*). These findings suggest that the vmPFC and dmPFC primarily represent motivational valence and contextual relevance rather than motor vigor. Their influence on action therefore likely acts indirectly through subcortical motivational circuits, particularly the VM and caudate nucleus, which in turn modulate motor cortical excitability. This dissociation supports hierarchical models in which prefrontal value representations bias downstream motivational and motor circuits without directly determining movement kinematics.

### Topographic organization of the human VM and striatum for motivational valence

We identified functionally distinct subregions within the human VM that recapitulate the topographic organization previously described in nonhuman primates (Matsumoto and Hikosaka, 2009). In macaques, dopamine neurons in the VM are spatially segregated according to motivational valence: ventromedial neurons, mainly within the VTA, respond preferentially to reward-predicting cues, whereas dorsolateral neurons, concentrated in the SNc, are excited by both reward and aversive cues. Our fMRI results revealed a homologous organization. The ventromedial VM was selectively activated during gain anticipation (Fig. 3*D*), whereas the dorsolateral VM exhibited comparable activation for both gain and loss contexts (Fig. 3*C*). Moreover, the dorsolateral VM encoded motivational salience independent of valence (Fig. 3*C*), consistent with the operational definition of motivational salience as the unsigned value of expected outcomes (Kahnt and Tobler, 2017).

This topographic organization was also observed in the striatum. The ventral striatum, including the NAc, exhibited overwhelmingly gain-specific activation (Fig. 2*D*). This gain specificity aligns with the dense connectivity between the NAc and the ventromedial VM and vmPFC (Haber and Knutson, 2010). In contrast, the caudate nucleus showed strong activation across both gain and loss contexts and sensitivity to motivational salience (Fig. 2*C*), paralleling the dorsolateral VM. Anatomically, the caudate nucleus receives projections from the dACC and dorsal prefrontal cortex and reciprocally connects with the SNc within the dorsolateral VM (Haber and Knutson, 2010). In nonhuman primates, the pregenual ACC, where approach- and avoidance-encoding neurons coexist (Amemori and Graybiel, 2012), projects preferentially to striosomes in the caudate (Eblen and Graybiel, 1995), which target SNc dopaminergic neurons (Crittenden et al., 2016) and have been causally implicated in approach-avoidance decision-making (Friedman et al., 2015). This corticostriatal pathway may provide an anatomical substrate through which valence-sensitive prefrontal signals influence motivational salience processing in the dorsolateral VM. The vmPFC transfers positive motivational valence to the NAc and the ventromedial VM. The caudate nucleus conveys motivational salience to the dorsolateral VM.

### Convergent mesocortical pathways for motivated action

An important aspect of the present study is the dissociation between the neural pathways governing the scaling of motor output strength and those governing movement initiation. Premovement activity in the dorsolateral VM and caudate nucleus was selectively related to subsequent force production (Figs. 4*E*, 5*C*–*E*), whereas the RT was associated with activity in a partially distinct set of striatal and cortical motor regions, including the NAc, putamen, and M1 (Figs. 4*D*, 5*C*,*D*). This dissociation suggests that motivational value is not conveyed to the motor system as a single uniform signal but is partitioned into components that differentially influence the strength and timing of action. Motivational valence appears to shape behavior primarily during upstream stages of this circuit. Consistent with our previous study (Sugawara et al., 2023), VM premovement activity was selectively associated with subsequent force production but not with RT (Fig. 4*D*,*E*), supporting its role in scaling motor output strength. Anatomical and physiological evidence from nonhuman primates shows that VM dopaminergic neurons project to the motor cortex via mesocortical pathways (Williams and Goldman-Rakic, 1998) and influence spinal motor circuits through multisynaptic pathways involving M1 (Suzuki et al., 2022). Notably, caudal VM dopaminergic neurons contribute more prominently to this VM–M1–spinal pathway than rostral VM neurons, indicating a rostrocaudal gradient in the VM's descending influence on the motor system. Given that caudal VM populations include neurons responsive to both reward and punishment, this organization suggests that the VM may serve as a critical output node through which motivational signals—irrespective of valence—are transmitted to corticospinal motor circuits. This direct VM to M1 pathway may extend the classical limbic–motor interface to humans. Whereas the original model in rodents emphasized a serial pathway through the NAc and ventral pallidum (Mogenson et al., 1980), our results raise the possibility that a more direct dopaminergic projection contributes to the scaling of force production.

In contrast to force production, RT was associated with a distinct neural substrate. RT was not correlated with VM premovement activity (Fig. 4*D*). Instead, it was related to activity in a subset of the NAc and in cortical motor regions, including M1 (Figs. 4*D*, 5*C*,*D*). Although the NAc does not project directly to M1 (Williams and Goldman-Rakic, 1998), M1 premovement activity correlated with both RT and peak grip force (Figs. 4*D*,*E*, 5*D*), suggesting that NAc motivational signals influence movement initiation through indirect pathways. These findings imply three potential pathways connecting motivation to movement initiation. First, the NAc may influence M1 through basal forebrain cholinergic neurons, which provide multisynaptic projections to M1 (Miyachi, 2005). Second, the NAc projects to the ventral pallidum, which innervates motor thalamic nuclei that could gate movement initiation (Haber et al., 1990). Third, motivational signals may reach motor circuits through ventral-to-dorsal striatal “spiral” connections (Haber et al., 2000), consistent with our finding that putamen activity is correlated with RT (Fig. 4*D*). These pathways may operate in parallel to translate motivation into movement initiation, distinct from the VM–M1 pathway, which selectively modulates force production.

### Clinical and translational implications

Understanding how motivation translates into motor output has clinical relevance. In Parkinson's disease, degeneration of SNc dopaminergic neurons leads to bradykinesia and reduced movement amplitude (Berardelli et al., 2001). Our findings suggest that impaired motivational modulation of force may arise from disruption of VM–motor pathways, whereas deficits in movement initiation may reflect dysfunction in parallel striatal circuits. Although speculative, this dissociation implies that therapeutic interventions may affect these motor parameters differentially depending on the targeted circuit. More broadly, these findings may guide motivationally informed rehabilitation to optimize motor performance.

### Methodological considerations regarding multiband acquisition

A methodological consideration concerns multiband EPI with a sixfold acceleration factor. High multiband factors can reduce the temporal SNR (TSNR) in central brain regions (Risk et al., 2018) and diminish sensitivity for detecting mesolimbic reward-related responses. In particular, effect sizes for gain anticipation in the NAc are reduced by more than half relative to single-band acquisition (Srirangarajan et al., 2021). Accordingly, our primary conclusions concern the spatial organization of responses within the VM and striatum rather than the absolute magnitude of the effects. Two considerations mitigate this limitation. First, peak coordinates of reward-related activation do not shift with acquisition protocol (Srirangarajan et al., 2021). Second, any loss of sensitivity would attenuate rather than inflate subcortical effect sizes. Moreover, the expected mesolimbic activations were still detected in the NAc and VM (Figs. 2*D*, 3*C*,*D*). Thus, the observed NAc and VM effects are unlikely to reflect the acquisition protocol alone. Future studies resolving finer subregional organization may use single-band protocols or lower multiband acceleration factors.

### Conclusion

The present study reveals that approach and avoidance motivations engage distinct prefrontal representations—the vmPFC for gains and the dmPFC for losses—that partially converge in the dorsolateral VM. Critically, the VM and striatum encode distinct aspects of motor output: the dorsolateral VM and caudate nucleus selectively predict grip force, whereas the NAc and putamen represent RT. These findings demonstrate a hierarchical circuit in which motivational valence is differentiated cortically, integrated subcortically, and transformed into motor output through parallel pathways that independently modulate movement initiation and force production.
